# Genetics of inherited peripheral neuropathies and the next frontier: looking backwards to progress forwards

**DOI:** 10.1136/jnnp-2024-333436

**Published:** 2024-05-14

**Authors:** Jevin M Parmar, Nigel G Laing, Marina L Kennerson, Gianina Ravenscroft

**Affiliations:** 1 Rare Disease Genetics and Functional Genomics, Harry Perkins Institute of Medical Research, Perth, Western Australia, Australia; 2 Centre for Medical Research, Faculty of Health and Medical Sciences, The University of Western Australia, Perth, Western Australia, Australia; 3 Preventive Genetics, Harry Perkins Institute of Medical Research, Perth, Western Australia, Australia; 4 Northcott Neuroscience Laboratory, ANZAC Research Institute, Concord, New South Wales, Australia; 5 Molecular Medicine Laboratory, Concord Hospital, Concord, New South Wales, Australia

**Keywords:** GENETICS, HMSN (CHARCOT-MARIE-TOOTH), NEUROGENETICS, NEUROMUSCULAR, NEUROPATHY

## Abstract

Inherited peripheral neuropathies (IPNs) encompass a clinically and genetically heterogeneous group of disorders causing length-dependent degeneration of peripheral autonomic, motor and/or sensory nerves. Despite gold-standard diagnostic testing for pathogenic variants in over 100 known associated genes, many patients with IPN remain genetically unsolved. Providing patients with a diagnosis is critical for reducing their ‘diagnostic odyssey’, improving clinical care, and for informed genetic counselling. The last decade of massively parallel sequencing technologies has seen a rapid increase in the number of newly described IPN-associated gene variants contributing to IPN pathogenesis. However, the scarcity of additional families and functional data supporting variants in potential novel genes is prolonging patient diagnostic uncertainty and contributing to the missing heritability of IPNs. We review the last decade of IPN disease gene discovery to highlight novel genes, structural variation and short tandem repeat expansions contributing to IPN pathogenesis. From the lessons learnt, we provide our vision for IPN research as we anticipate the future, providing examples of emerging technologies, resources and tools that we propose that will expedite the genetic diagnosis of unsolved IPN families.

## Introduction

 Inherited peripheral neuropathies (IPNs) are a group of inherited diseases affecting the peripheral nervous system with a wide range of symptoms, clinical severity and causes.^1 2^ IPNs show all modes of inheritance (autosomal dominant, autosomal recessive, X-linked and mitochondrial) and are grouped depending on the peripheral neurons involved: hereditary motor and sensory neuropathies, hereditary motor neuropathies (HMN) and hereditary sensory and autonomic neuropathies (HSAN). The most common form is hereditary motor and sensory neuropathy (also known as Charcot-Marie-Tooth (CMT) neuropathy) with a prevalence of 1 in 2500.^1 3 4^ CMT is divided into demyelinating (CMT1), axonal (CMT2) or intermediate (CMTi) depending on the pathophysiology. In CMT1, the primary pathology is degeneration of the myelin sheath leading to nerve conduction velocity (NCV) reduction, whereas axon degeneration, leaving NCV intact, is the primary pathology in CMT2.^1 2^ In CMTi, NCVs overlap both CMT1 and CMT2, and may show pathology of both CMT1 and CMT2.^2 4 5^ Patients with IPN may present with features additional to neuropathy, including metabolic or mitochondrial dysfunction, neurodegenerative disorders including hereditary ataxias, and leukodystrophy; or may be part of a complex multisystem disorder.^25^,^7^

Genetic diagnoses of IPNs are mostly attributed to single nucleotide variants (SNVs), and structural variants (SVs), including copy number variants (CNVs), in a small number of genes that have been known for many years: *GJB1*, *MFN2*, *MPZ, PMP22*.^2^,^5^ The most common disease-causing variant is a 1.5 Mb duplication resulting in three copies of *PMP22*, while SNVs are the most common disease-causing variants in *GJB1*, *MFN2* and *MPZ*. However, pathogenic variants in over 100 IPN-associated genes and loci have been published.^2^,^46^ Due to the large number of known IPN-associated genes, the broad phenotypic spectra associated with individual genes and the overlap of genotype-phenotype boundaries, massively parallel sequencing (MPS) technologies have been adopted in both diagnostics and research.^2 4 10^ Diagnostic targeted gene panels have reduced cost and workforce time,^11 12^ while research whole-exome sequencing (WES) and whole-genome sequencing (WGS) have powered gene discovery for molecularly unsolved patients with IPN.^1113^,^16^ In the last 10 years, pathogenic or likely pathogenic variants in 73 genes and 2 genetic loci were associated with IPNs (online supplemental table 1). These include genes and loci that (1) were not previously associated with disease, or (2) were associated with another disease or form of IPN prior to 2012, but have since been newly associated with a form of IPN. Pathogenic variants in IPN-associated genes identified prior to 2012 are reviewed elsewhere.^3^,^59^

Despite the increase of reported disease-associated genes through MPS diagnostics, improved bioinformatic analyses and functional genomics, over 50% of patients in some IPN cohorts remain genetically unsolved.^1012 15 17^,^19^ Multiple factors likely contribute to this diagnostic gap. These include: (1) further pathogenic variants in novel IPN genes that remain to be identified, (2) difficulties in reclassification of variants of unknown significance (VUSs) in known IPN-associated genes, and (3) identification and interpretation of non-coding variants and SVs. Other bottlenecks in diagnosis include insufficient data sharing and a lack of high-throughput functional validation of candidate pathogenic variants.^20^ Additionally, some patients clinically diagnosed with IPN may have non-genetic causes or non-Mendelian disease, including digenic inheritance.^5 16 17 21^ Families without a molecular diagnosis face a convoluted and expensive ‘diagnostic odyssey’.^22 23^

This review explores the evolving genetic landscape of IPNs over the last 10 years including the identification of pathogenic variants in novel genes, disease-causing SVs and short tandem repeat (STR) expansions. By extrapolating from challenges overcome to the challenges that still remain, we provide an outline for tackling the ‘The Final Frontier’ of the missing heritability of IPNs, enabling a positive trajectory towards accurate genetic diagnoses for the many families with IPN still to be solved.

## Looking back at the last 10 years: lessons learnt from recent gene discoveries

Of the 73 genes and 2 loci identified in the last decade (online supplemental table 1), a large percentage have been associated with CMT, but there has also been an appreciable increase in HSAN-associated and HMN-associated genes.

Over 30 of the 73 genes are associated with multiple phenotypes. These include: (1) other neuropathy subtypes (eg, *HINT1*); (2) other rare neurogenetic disorders (eg, *CNTNAP1*); and (3) more common disorders, for example, intellectual disability (eg, *NEMF*) (online supplemental table 1).^23 5^,^7 24^

**Figure 1:**
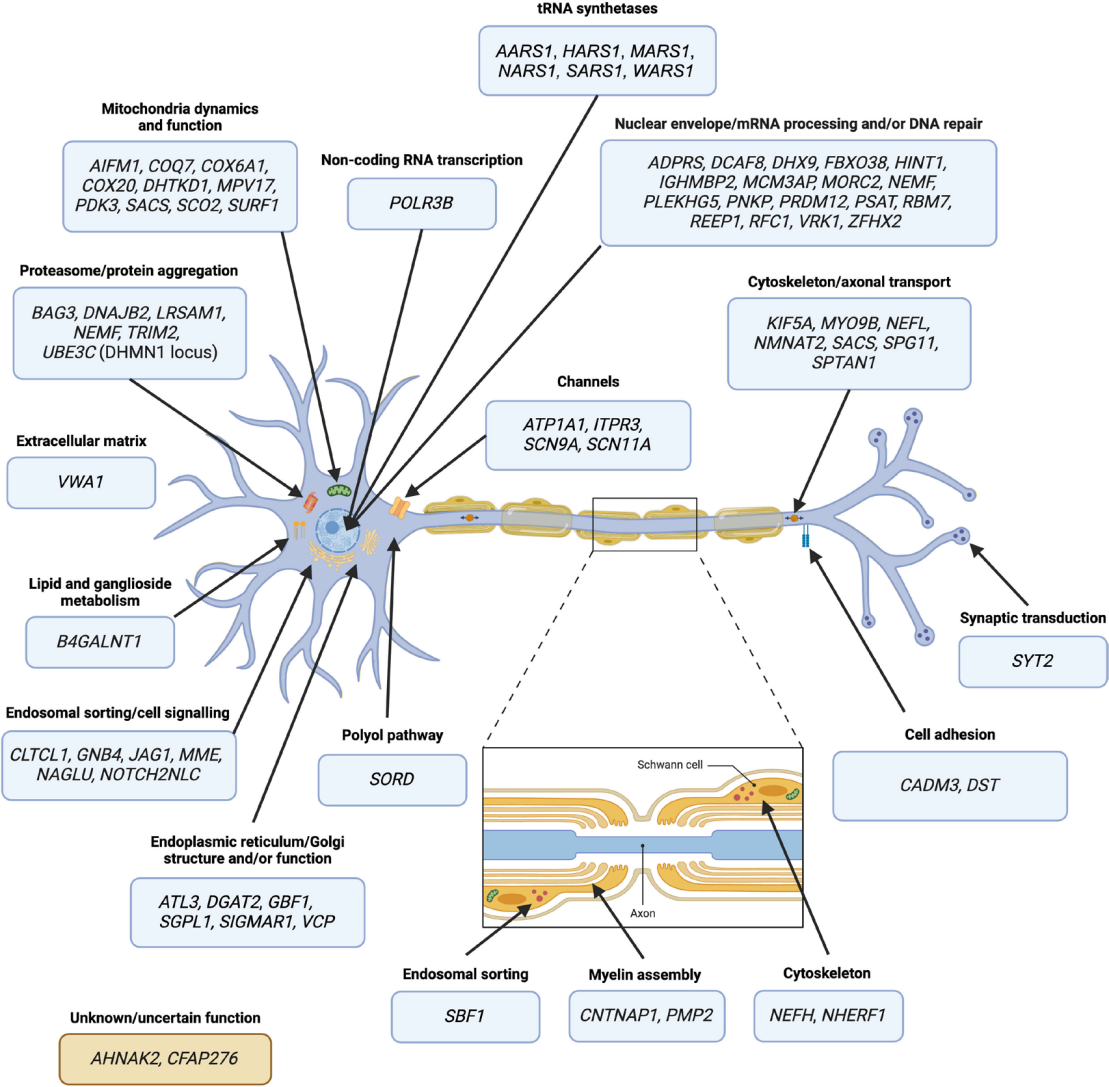
Pathomechanisms of IPN-associated genes identified since 2012. Genes are categorised according to their principal function, pathway and/or their proposed pathomechanism. Note: the CMTX3 locus is not included. Illustration created with BioRender. IPN, inherited peripheral neuropathy; mRNA, messenger RNA; tRNA, transfer RNA.

Identifying the 73 genes has expanded the biological pathways involved in IPN pathogenesis (figure 1), including extracellular matrix composition, lipid and ganglioside metabolism, the polyol pathway and endosomal sorting.^36^,^9^

The following IPN gene discoveries illustrate how challenges in finding the molecular causes of IPNs were overcome and provide lessons to tackle the still missing genetics of IPNs.

### *MME:* Reduced expressivity/penetrance complicates molecular diagnoses

The *MME* gene encodes neprilysin, a membrane metalloendopeptidase.^25 26^ In 2016, pathogenic variants in the gene were reported to cause both dominant spinocerebellar ataxia (SCA) with neuropathy (SCA43),^25^ and recessive CMT (AR-CMT2T).^26^ In AR-CMT2T, bi-allelic *MME* variants cause peripheral neuropathy with an average age of onset of 45 years.^27^

Heterozygous *MME* variants have also been reported as a relatively common cause of milder, later-onset CMT with both reduced penetrance and semidominance.^25^,^28^ However, the association of heterozygous *MME* variants with autosomal dominant CMT has been controversial,^4 27 28^ with suggestions that these variants do not cause disease, but are possibly risk alleles increasing susceptibility to disease.^4 27^ Reduced penetrance has also been reported for variants in more common IPN-associated genes, including *PMP22* and *MFN2*.^29^,^32^ *MME* highlights the importance of considering reduced penetrance and risk alleles when interpreting the pathogenic impact in unsolved patients with IPN.

#### *SORD and VWA1:* Sequencing and/or mapping difficulties hinder disease gene discovery

Pathogenic variants in *SORD*, a gene encoding sorbitol dehydrogenase, were recently identified as a frequent cause of CMT2 and distal HMN (dHMN).^33^,^36^ With *SORD* variants now recognised as one of the most common causes of recessive IPN, and the c.757delG variant having a carrier frequency of ~3:1000,^33^ why was the causative link not established earlier? Cortese *et al*^33^ speculate that analysis was complicated by the presence of the *SORD2P* pseudogene, causing mis-mapping of the short-read sequencing reads. Grosz *et al*^37^ later bypassed the short-read issues by using long-read sequencing to categorically identify biallelic *SORD* variants. There are many other genomic regions that remain difficult to interrogate. For example, Ebbert *et al*^38^ describe >36 000 dark regions in 6054 genes that are inadequately assembled or aligned with data generated from short-read technologies. These issues are partially being overcome by long-read technologies. Similar read mapping issues may be hampering the identification of pathogenic variants in other novel IPN genes.

In 2021, homozygous or compound heterozygous loss-of-function *VWA1* variants were reported as a cause of recessive dHMN with myopathic features (neuromyopathy).^39 40^ The most common disease-causing variant reported was a 10 bp duplication (c.62_71dup10) resulting in a frameshift (p.Gly25ArgfsTer74).^39 40^ The c.62_71dup10 variant is the most frequent *VWA1* variant in gnomAD (minor allele frequency 0.06%,^39^ rising to 0.091% in non-Finnish Europeans) with no reported homozygotes.^40^ The reference genome contains two repeats of the 10 bp motif. Pathogenic expansion to three repeats appears to be stable.^40^ Given the variant’s enrichment in patient cohorts and frequency in gnomAD, it is interesting that it was not identified earlier as a cause of neuropathy. Pagnamenta *et al*^40^ suggest that the high guanine-cytosine (GC) content of the region likely hindered the identification of the duplication. Undiagnosed patients with predominant axonal motor neuropathy and suspected autosomal recessive inheritance should be reanalysed to screen for the disease-causing duplication (and other variants) in *VWA1*.

### Structural variation in IPN

SVs are DNA re-arrangements >50 bp and can include unbalanced DNA duplications and deletions (CNVs), balanced re-arrangements without dosage alterations (insertions, inversions and translocations) or a combination of these (complex SVs).^41 42^ SVs may result in disrupted chromosomal organisation and three-dimensional (3D) genome architecture.^41 42^ The importance of SVs in Mendelian disorders is increasingly recognised.

In the early 1990s, CMT1A, the most common subtype of CMT, was shown to result from a 1.5 Mb tandem duplication of chromosome 17p11.2.^43^,^48^ The reciprocal deletion was later shown to cause hereditary neuropathy with liability to pressure palsies.^49^ Whole or partial gene duplications or deletions have since been described for other CMT genes, including *GJB1*, *MFN2*, *MPZ* and *NDRG1* (reviewed in a study by Cutrupi *et al*^41^). Recently, a 650 bp deletion of the *PMP22* 3’ untranslated region (UTR) was reported for a severe case of CMT1 negative for the CMT1A duplication.^50^ Determining the contribution of SVs to unsolved IPNs is challenging since there are many SVs in each genome, including CNVs, translocations, inversions and complex rearrangements.^51 52^ Successful strategies to investigate SVs have included: (1) linkage analysis in large unsolved IPN families followed by targeted WGS, (2) examining known IPN-associated genes, including upstream and downstream regulatory regions and (3) analysis of parent–child trio WGS data.

Two SVs causing IPN are discussed below. In both cases, linkage analysis mapped the disease locus in the family and short-read WGS identified the genomic rearrangement. The families had previously undergone targeted diagnostic gene panel testing and WES, but this failed to identify a causative variant.

#### CMTX3

In 2016, Brewer *et al*^53^ reported an SV responsible for an X-linked form of CMT (CMTX3). The disease locus was mapped to a 5.7 Mb interval at Xq26.3-q27.2.^54^ Short-read WGS identified a 78 kb insertion of chromosome 8q24.3 into a gene desert at Xq27.1, in the CMTX3 region. Three families were originally identified with this insertion but are likely related through a common founder.^53 55 56^ A partial duplication of *ARHGAP39* (exons 1–7) within the 78 kb insertion suggested this could be a candidate gene acting through aberrant gene dosage.^53^ However, there was no difference in *ARHGAP39* expression between patient and control lymphoblasts. Interestingly, there was a three-fold increase in *FGF13* expression,^53^ which lies upstream of the SV. This supports *FGF13* as a candidate disease gene and its dysregulation as the disease mechanism, although *FGF13* transcription remains to be determined in neurons.

#### DHMN1

A complex insertion at the DHMN1 locus on chromosome 7q34-q36 was reported by Drew *et al*.^57^ The pathogenic SV involved a 1.35 Mb region of 7q36.3 being inverted and inserted within the DHMN1 locus at 7q36.2. Five protein-coding genes with associated promoters and enhancers (*LMBR*, *LOC389602*, *MNX1*, *NOM1*, *RNF32*), a partial copy of the *UBE3C* gene and three long non-coding RNAs (*LINC01006*, *LOC285889*, *MNX1-AS1*) were introduced.^57^ Recently, it was reported that the DHMN1 genomic rearrangement causes the formation of a novel gene intergenic fusion transcript (*UBE3C-IF*) via the *UBE3C* partial transcript, incorporating a pseudo-terminal exon from an intergenic sequence within the DHMN1 locus.^58^ Analysis of iPSC-derived patient motor neurons suggests *UBE3C-IF* produces a dominant negative effect and significantly reduces wild-type UBE3C protein levels.^58^ The *UBE3C-IF* in motor neurons identifies a new disease mechanism underlying axonal and motor neuron degeneration.

### STR expansions in IPN

STRs are increasingly implicated in IPNs. STRs are 1–6 bp long DNA sequences repeated multiple times in tandem.^59 60^ Around 3% of the human genome is composed of STRs.^59 60^ They have mutation rates 10 to 100 000 times higher than other genomic regions.^61^ STRs have previously been implicated in a range of neurological disorders,^59 60 62^ however their contribution to IPNs has not been extensively described. Recent investigations have identified STR expansions causing neuropathy or syndromic disorders with neuropathy included in the phenotype.

#### FGF14

GAA expansions in *FGF14* were recently identified as a common cause of late-onset dominant ataxia worldwide.^63 64^ Mild peripheral neuropathy was reported as part of the phenotype in approximately 11–25% of patients, but was not a core feature.^63 65 66^ Some studies reported no neuropathic features in GAA-*FGF14* positive cases.^64 67 68^ Whether IPN is a consistent feature of GAA-FGF14-related ataxia remains to be clarified.

#### NOTCH2NLC

In 2019, three groups reported GGC repeat expansions in *NOTCH2NLC* causing neuronal intranuclear inclusion disease (NIID).^69^,^71^ NIID is a neurodegenerative disorder characterised by multiple clinical features, including peripheral neuropathy.^69^,^71^ The GGC STR lies in the *NOTCH2NLC* 5’ UTR.^69^,^71^ Familial and sporadic NIID cases had heterozygous *NOTCH2NLC* expansions, with either autosomal dominant or de novo inheritance.^69^,^71^ Notably, patients presenting with weakness had GGA interrupts within the GGC expansion,^69^,^71^ suggesting the GGA motif modifies the NIID phenotype.

#### RFC1

Peripheral neuropathy is one hallmark feature of cerebellar ataxia, neuropathy and vestibular areflexia syndrome (CANVAS): a multisystem, adult-onset, slowly-progressive disorder with autosomal recessive inheritance.^72^,^74^ In 2019, biallelic pentanucleotide AAGGG expansions in the second intron of *RFC1* were identified as causing CANVAS.^72^ Pathogenic expansions range from 400 to 2750 repeat units.^72^,^76^ Biallelic *RFC1* expansions are relatively common in patients with neuropathy: 14.5% and 34% of genetic diagnoses in two independent chronic idiopathic axonal polyneuropathy cohorts,^77 78^ and 25.3% and 72.7% of selected patients with HSAN.^79 80^ Since the initial discovery, additional polymorphic and pathogenic repeats, and loss-of-function SNVs have been identified.^7576 81^,^84^

#### A possible role for other STR expansions in IPN

The findings above suggest STR expansions likely contribute to unsolved IPN cases. Shorter GAA expansions at the *FGF14* locus (~250 repeats) are reported to be associated with reduced penetrance and variable meiotic stability in late-onset ataxia, which likely hampered the initial identification of the disease.^63 64^ The discovery of additional pathogenic STR expansions for IPN may be similarly masked by phenotypic and genetic factors. Discovery is also confounded by the limitation of bioinformatic tools to align, detect and accurately size STR expansions generated from short-read data. New tools to detect novel STR expansions, for both short-read^85^ and long-read^62^ WGS are being developed and will aid further discovery of STR-related IPNs.

### Non-Mendelian inheritance in IPN

IPNs have traditionally been considered Mendelian disorders, with the bulk of disease gene discovery and diagnostics focused on monogenic disease.^2^,^46^ However, suspected digenic inheritance for IPN has been reported.^19 21^ Several reports have involved variants in *MFN2* and *GDAP1*, where the cumulative effect of these variants has resulted in severe IPN.^86^,^89^ Cassereau *et al*^86^ reported a patient with severe CMT harbouring previously observed pathogenic homozygous loss-of-function *GDAP1* and heterozygous missense *MFN2* variants. Similarly, Vital *et al*^87^ identified compound heterozygous missense *GDAP1* and loss-of-function *MFN2* variants in the proband and affected daughter with severe CMT. More recent studies have shown non-Mendelian inheritance, with concomitant *MFN2* and *GDAP1* variants resulting in disease.^88 89^ Importantly, other family members in the above cases harbouring a heterozygous variant in either *GDAP1* or *MFN2*, were asymptomatic, presented with a subclinical phenotype, or had mild IPN.^86^,^89^

For some of the most challenging unsolved IPN families, identifying causes beyond monogenic disease and the possibility of complex causes is another feasible approach.^21 90 91^ True digenic causes of genetic disease are challenging to identify as the causative variants may be common in the population or may individually only confer a small effect on gene function.^21 90^ Furthermore, to classify a gene pair as truly digenic requires both sufficient genetic and functional evidence.^92^ Considering digenic/oligogenic inheritance to identify causative gene variants highlights an important paradigm shift for IPN gene discovery. Centralised databases of known digenic/oligogenic causes of disease and criteria for identifying digenic/oligogenic disease are being developed.^92 93^

## The next 10 years: strategies to address the missing heritability of IPNs

**Figure 2:**
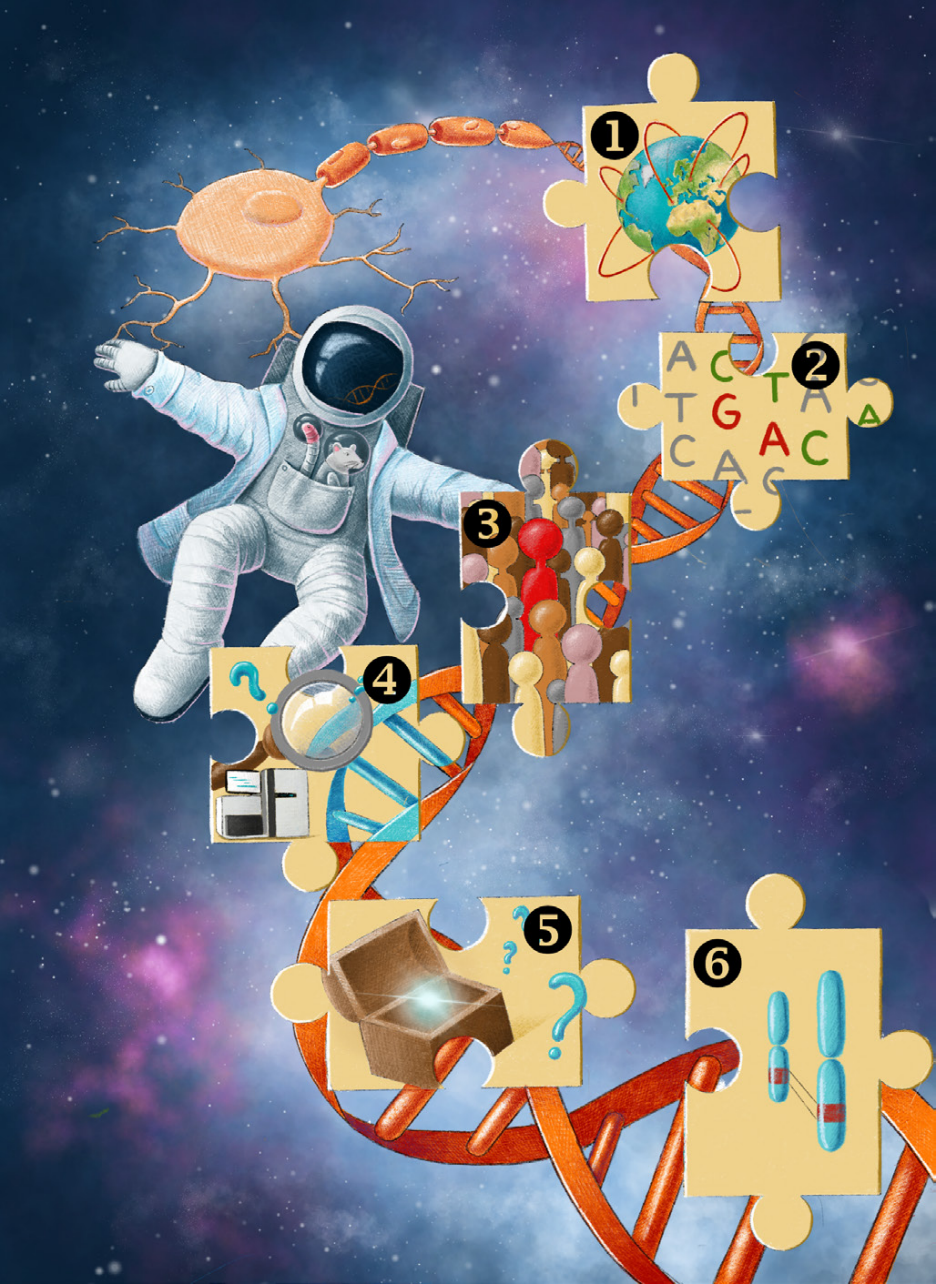
Strategies to tackle ‘The Final Frontier’ for the molecular diagnosis of inherited peripheral neuropathies. (1) Many discoveries have and will continue to be facilitated by global networks, databases and variant sharing. (2) Variant interpretation remains an obstacle. With concerted efforts to perform saturation mutagenesis and provide functional scores for all variants, the proportion of variants of uncertain significance may be reduced considerably. (3) Challenges remain around making a molecular diagnosis in n=1 cases and families. As we delve deeper into ‘The Final Frontier’ we need to pursue functional genomics, including studies in model organisms, to push these n=1 families over the line for diagnostic reporting. (4) Emerging genomic tools including long-read sequencing and optical genome mapping, new informatic approaches and reference data sets will greatly enhance our identification of the missing heritability of the inherited peripheral neuropathies. (5) Currently, much of the non-coding genome remains a black box; identifying regions of the genome critical to gene regulation and function and non-coding pathogenic variants will facilitate molecular diagnostics. (6) Sequencing, mapping and interpreting structural variants within the genome is difficult with current technologies. However, structural variants are likely to explain a proportion of the missing heritability of inherited peripheral neuropathies and should be prioritised for molecular diagnosis of unsolved patients with neuropathy.

Although pathogenic variants in over 100 genes have already been identified for IPN, many more are likely yet to be identified as the rate of Mendelian gene discovery is not decreasing.^20^ In addition, unsolved patients with IPN will require more rigorous interrogation beyond just the exome to identify novel disease-causing variants in the non-coding region of known IPN-associated genes (eg, deep intronic variants that alter splicing).^94^ We describe six strategies we project will help meet the challenge of the missing heritability of genetically unsolved IPN families over the next decade (figure 2).

### Strategy 1: the importance of patient and population databases and open data sharing

It is well recognised that data sharing and international consortia are crucial in identifying the causes of rare genetic diseases.^95^

Databases that specifically include patients with IPN have aided cohort studies and gene discoveries for IPNs. The online GENESIS database^96^ has facilitated the identification of approximately one-third of the 73 genes and 2 IPN loci described in this review (online supplemental table 1), based on an examination of publications reporting these IPN-associated genes and loci. Similarly, the Broad Institute’s open-source, web-based tool, *seqr*, has contributed to the discovery of over 300 novel disease genes, including over 10 novel IPN-associated disease genes.^97^ Candidate disease genes can also be submitted to match-making programmes (eg, GeneMatcher),^98^ enabling connections to other research groups with patients harbouring variants in the same gene. This can assist in confirming variants in the candidate gene as disease-causing.

Capturing global diversity is essential for larger population databases. Genetic data from individuals of European ancestry continues to be disproportionately represented in population databases.^99^,^101^ As a result, genetic variation unique to other geographical or ethnic minorities is under-represented, or completely missed, but could hold key insights into population-specific disease prevalence. Headway is being made to represent global genetic diversity in reference genomes (see Strategy 4), and in population databases. For example, gnomAD is currently the most widely used reference population database,^100 101^ with the latest release (v4) including data from over 800 000 individuals. This release includes data from individuals of multiple under-represented ethnic groups but constitutes less than 25% of the total data. The contribution of ethnic minorities to rare disease genetic research cannot be understated, and concerted global efforts are a work in progress to realise the benefits of genomic medicine for all individuals.

### Strategy 2: re-classifying variants of uncertain significance

Many variants identified in known disease genes are classified as VUS and have retained the classification for years until sufficient evidence for their pathogenicity accumulates to satisfy the American College of Medical Genetics guidelines.^102^ The stringent guidelines mean variants in novel disease genes are only reported as a VUS until the gene is published.^102^

Another limitation of VUS re-classification is the absence of a second individual and/or family with the same variants or different variants in the same candidate gene. This is an issue especially for ultra-rare IPNs where candidate variants are often private (see Strategy 3), leaving the patient and their family at an impasse if the guidelines are used strictly.

Insufficient functional evidence to classify a variant as likely pathogenic or pathogenic is another significant bottleneck in variant re-classification.^20 103^ In silico predictions are inadequate substitutes for experimental analysis and validation. Candidate novel disease variants and/or genes frequently require functional investigation in vitro and/or in vivo to confirm pathogenicity. The relevant functional genomic test is needed as: (1) diagnostic reporting requires knowledge of the pathological function of a protein, and (2) defining appropriate cut-offs to categorise the pathogenic or benign status of a gene variant may be difficult. Brnich *et al*^104^ have provided a four-step framework to interpret functional evidence with the intention to create global collaborative efforts and fast-track VUS re-classification.

Cost and time constraints make ad hoc functional analysis of VUS difficult for providing prompt benefits to patients.^103 104^ Multiplexed assays for variant effect offer one approach to functionally investigate thousands of VUS in a single experiment.^103 105^ Another approach is deep mutation scanning, or saturation mutagenesis, for all possible coding variants, allowing in vitro pathogenicity prediction.^106 107^ This method has been performed for limb-girdle muscular dystrophy,^106 107^ and could be applied to IPN-associated disease genes. Ideally, such assays would show large differences in functional effect between benign and pathogenic variants. Current obstacles to widespread use include reproducibility, scalability, complexity and cost.^103^

Animal models also provide strong evidence to assess variant impact, particularly for findings of n=1. *Caenorhabditis elegans* models have been informative when investigating variants causing axonal neuropathies. Modelling the *PDK3* p.Arg158His pathogenic variant demonstrated synaptic transmission and neuronal deficits.^108^ However, as *C. elegans* does not have myelin, the model is not useful for demyelinating neuropathies. Rodent models may provide valuable functional information because they can closely recapitulate neurogenetic disease phenotypes.^109^ Nevertheless, to increase the diagnostic yield of IPNs, high-throughput, informative functional genomics that can conclusively categorise variants as pathogenic or benign are needed.

Periodic re-analysis of existing genomic data is important. Re-analysis can increase diagnostic rates by approximately 15%.^17 18^ Each new discovery, reference genome, tool, pipeline and functional assay provides an ideal opportunity to re-investigate the genome of unsolved patients to identify the disease-causing variant(s). Automated re-analysis and artificial intelligence tools are being developed to reduce the labour-intensive nature of data re-analysis.^110^

For patients with recessive forms of IPN, the diagnostic journey may come to a halt when only a heterozygous variant in a known IPN-associated gene is identified. But, the variant cannot fully explain the phenotypic manifestation of IPN, often remaining as a VUS. In such cases, investigation of digenic/oligogenic causes of disease could uncover a second hit. This may involve WGS of patient DNA that initially underwent WES to identify non-coding variants in known genes, and/or long-read sequencing or optical genome mapping (OGM) of patient DNA to identify SVs or STR expansions. Regardless, such manual research analysis is currently time-intensive and labour-intensive, highlighting the need for improved bioinformatic algorithms to discover novel digenic/oligogenic causes of disease. Machine learning approaches hold the potential to streamline the exploration of digenic causes of IPN.^111^

### Strategy 3: the ultra-rare IPNs, including n=1

CMT and related disorders are considered rare diseases as defined by the National Organisation of Rare Diseases.^112^ There are different definitions of ultra-rare disorders,^113^,^115^ but in this review we use ultra-rare for genes in which the variants are private or in <30 patients worldwide.

Ultra-rare IPNs may account for a significant proportion of the missing heritability of IPNs. Most studies have focused on the more common IPNs and their genetic causes.^5^ The identification of gene variants for ultra-rare IPNs has been accelerated by MPS, databases of human variation and bioinformatic pipelines that enable the identification and classification of genes with extremely rare variants.^53 57 116 117^ Of the IPN-associated gene discoveries since 2012 (online supplemental table 1), 46% would be considered ultra-rare. For example, SVs at the CMTX3 and DHMN1 loci are considered ultra-rare causes of IPN as they have currently only been identified as the cause of disease in <30 individuals worldwide.

Diagnosis of patients with ultra-rare disorders is confounded when novel gene candidate variants are categorised as VUS. This confuses patients and clinicians due to the diagnostic uncertainty, leaving the path towards clinical management unclear.^23 103^ Diagnostic laboratories cannot report variants if they are found in only one family.^102^ Additionally, publishing cases of n=1 without functional evidence supporting the association of the novel variant or gene with the disease is challenging (see Strategy 2). Functional genomic assays will have to be developed for each ultra-rare disease gene.^103 104^ Regardless, we anticipate that over the next 10 years, reclassification of ultra-rare IPNs to rare IPNs is likely to occur at a greater rate as the field identifies more individuals with variants in the ultra-rare genes.

### Strategy 4: widespread adoption of new sequencing and mapping technologies

Further IPN-associated gene discovery will require strategies that overcome the limitations of current short-read MPS techniques and bioinformatic pipelines. Short-read MPS is useful for deep coverage of SNVs in non-repetitive regions. However, short reads of ~150 bp map poorly to complex, high GC content and repetitive regions. Consequently, accurate detection of STR expansions, SNVs in repetitive regions and CNVs is decreased.^23 118^ Additionally, the presence of pseudogenes can restrict the accurate identification of potentially pathogenic variants using short-read MPS.^33 37^ Emerging technologies and complete human reference genomes will aid interrogation of poorly mapped or missing regions of the current reference genome and facilitate disease gene discovery and molecular diagnosis.

#### Long-read sequencing

By sequencing DNA fragments up to >200 kb in length,^119^ long-read sequencing technologies (Oxford Nanopore Technology (ONT), and Pacific Biosciences) generate whole-genome data suitable for de novo assembly and analysis of repetitive regions, CNVs and SVs.^119 120^ The accurate genomic contextual information from long-reads enables reliable SV and STR expansion calling, and can assist with mapping the splicing profile of genes using transcriptomics.^120 121^ Recent studies have demonstrated the value of long-read sequencing for disease gene discovery and diagnostics,^62 70 121 122^ including the mis-mapping of *SORD* sequencing reads to the pseudogene *SORD2P*,^37^ and resolving GC-rich regions to identify the disease-causing repeat expansion in spinocerebellar ataxia type 4.^123^ Oxford Nanopore’s *ReadUntil* selective adaptive sequencing can provide enriched sequencing depth for any selected STRs,^62^ and may therefore offer a more cost-effective analysis of genome-wide known STR expansions.

#### Optical genome mapping

OGM technologies, such as the Bionano *Saphyr* platform, allow for unbiased, genome-wide identification of SVs and large STRs, including uncovering SVs in patients where short-read sequencing and microarray analyses were negative.^118 124^ OGM has been used to detect D4Z4 repeat deletions for diagnosing facioscapulohumeral muscular dystrophy.^125^ OGM, used in parallel with short-read MPS, has the capacity to identify missed pathogenic events during patient re-analysis, as achieved for inherited retinal diseases.^126^ OGM and long-read sequencing can also facilitate the identification of complex SVs. For example, Fazal *et al*^117^ identified a homozygous GCA repeat expansion nested within a 5’ UTR quadruplication event in *GLS* in a family with an ultra-rare disorder.^117 127^

The input DNA for OGM is an order of magnitude larger (N50: ~250 kb) than that used for ONT (10–20 kb) and requires specialised DNA preparation from fresh samples.^124 126^ Thus, OGM may be better at discerning large SVs, and could complement WGS data in screening for SVs, finding much-needed answers for unsolved patients with IPN.

#### Transcriptomics and proteomics

Short-read or long-read RNA sequencing (RNA-seq) are alternative techniques for examining genetic or epigenetic changes. However, this approach may require sequencing appropriate tissues (eg, nerve) to yield relevant data. RNA-seq of affected tissues, combined with MPS, may offer an avenue to investigate somatic mosaicism, particularly for patients who show asymmetry in their clinical presentation.^128^ Long-read transcriptomics will offer unparalleled potential to examine full-length messenger RNAs (mRNA) for altered gene expression, mRNA processing and aberrant splicing.^121^ For example, Clark *et al*^121^ employed ONT to characterise the splicing profile of *CACNA1C*, a psychiatric risk gene. The diversity of the human transcriptome is increasingly apparent, and long-read transcriptomics has emerged as an effective tool to address this.^121^ Miller *et al*^129^ developed a workflow for long-read proteogenomics, highlighting that protein isoform diversity, driven by alternative splicing, is much more prevalent than previously realised.

#### Improvement of reference genomes

The GRCh38 reference genome assembly is currently generally used as the standard human genome.^130^ However, GRCh38 contains over 150 Mb of undetermined sequence, including ribosomal DNA, pericentromeric and subtelomeric areas, and recent segmental duplications.^131^ To address these significant gaps in the genome, Nurk *et al*^131^ used long-read and ultralong-read sequencing technologies to assemble a uniformly homozygous Telomere-to-Telomere (T2T)-CHM13 reference assembly. The T2T-CHM13 reference increased the number of known genes from 60 090 to 63 494, and the percentage of repeats in the genome from 51.9% to 53.9%.^131^ Compared with the GRCh38 assembly, the T2T-CHM13 reference improved SNV and SV calling through increased coverage and copy number resolution of many loci, and a reduction in alternate or decoy contigs.^52^ In addition, false-positive SV calls were reduced and structural errors resolved.^52^

The recent release of the draft human pangenome heralds an exciting change in the genome reference landscape, providing a new lens for unravelling human genetic diversity and future disease gene discovery and diagnostics.^132 133^ The Human Pangenome Reference Consortium seeks to represent the cumulative diversity of the human species through the WGS of 350 index individuals and their parents by the end of 2024.^132^ Key to its completion will be the principle of selecting the remaining genomes that represent diversity, equity and inclusion, with special attention to under-represented ancestries. For instance, the pangenome could incorporate findings from ONT sequencing of 121 Indigenous Australians across four remote Aboriginal communities.^134^ Findings included an abundance of SVs and CNVs, comprised of tandem repeats or mobile element sequences, with up to 62% not previously annotated.^134^

#### De novo assembly

De novo variants are another source of variation that could explain part of the missing heritability of IPNs. De novo variants arise in the germline post-fertilisation, through multiple mechanisms.^135^ Dozens of de novo SNVs and indels are reported per individual,^102^ with larger de novo STR expansions and SVs reported at a much lower rate.^131 135 136^ But, these rates have been estimated using short-read sequencing, which is problematic when sequencing repetitive regions, high GC content regions or large SVs.^131 135 136^ As a result, the true de novo mutation rate is likely underestimated.^38^

To accurately determine the rate of de novo variation, familial long-read sequencing was used for de novo assembly of genomes in a family with autism.^135^ When compared with short-read data, long-read sequencing increased the discovery of de novo SNVs and indels by 20%.^135^ Discovery increased by an additional 5% when mapped to the T2T-CHM13 reference.^135^ Expanding this, Ebert *et al*^136^ constructed fully phased diploid genome assemblies for 35 individuals from diverse ethnic backgrounds. The computational pipeline incorporated both long-read and single-cell template strand sequencing methods to generate de novo assemblies.^136^ This method uncovered over 105 000 SVs, of which 68% were not identified with short-read sequencing methods.^136^ De novo assembly is thus a powerful method to uncover candidate disease variants and identify novel causes of disease in genetically unsolved patients with IPN.

### Strategy 5: shedding light on the non-coding regulatory genome

In the next 10 years, an area of increased focus for IPN research will likely be the non-coding genome.^20 137^

The non-coding genome refers to the 98% non-protein-coding portion of the genome, once believed to have no obvious purpose.^137^ Much of the non-coding genome is reported to be functional (such as repeat elements, enhancers, promoters, non-coding RNAs and distal regulatory sequences) and is predicted to harbour variants that cause genetic disease.^20 137^ For example, 5’ and 3’ UTR *GJB1* variants were identified as a cause of CMTX1.^138^

Identification and interpretation of regulatory regions and variants still remain a significant challenge, due to difficulties in aligning these regions with short-read WGS. However methods are being employed to better understand the regulatory landscape, including ATAC-seq, Hi-C, cap analysis of gene expression, and ChIP-seq (discussed further in studies by French and Edwards, and Ellingford *et al*^137 139^). Insights from fundamental gene regulation and biology will inform the interpretation of non-coding, likely regulatory variants in IPN.

There are however also difficulties in reporting of variants in non-coding regions. Recommendations for interpreting non-coding variants have been published but they have yet to be adopted as a governing set of guidelines.^139^

Regardless, our understanding of the non-coding genome is incomplete. Identifying pathogenic variants outside the coding genome will help bridge the diagnostic gap and greatly assist in resolving the missing heritability of IPNs.

### Strategy 6: improving identification and interpretation of structural variants

When investigating the cause of disease for unsolved patients with IPN, SVs are usually not prioritised for investigation. A key limitation has been the completeness of the reference genome. Aligning short-read sequencing data to commonly used reference genomes (GRCh37 and GRCh38) can create false duplicated and collapsed regions, resulting in low and high signal regions, and misalignments.^140^ Consequently, the rate of false-positive SV calls increases.^140^

Another challenge posed is predicting the functional consequence of SVs. In silico prediction of the consequences of SVs is more difficult than for SNVs since the impact of SVs on gene expression and protein structure is not well understood and is harder to interpret.^51^ Inversions or duplications can be particularly difficult to interpret. Are the genomic consequences of such SVs only at DNA breakpoints or due to the genomic re-arrangement impacting the 3D genome? What are the consequences of deletions or duplications involving gene UTRs or introns? Relevant functional studies are required for candidate disease-causing SVs. There has been some success in the identification of disease-causing SVs for IPNs (eg, CMTX3 and DHMN1). However, current novel SVs identified are restricted to single families or are only present in a few individuals, suggesting that SVs are contributing to ultra-rare IPN.

There are several strategies for improving SV detection and interpretation that we envision will address the missing heritability of IPNs:

1. Long-read sequencing alignment and/or OGM to resolve complex and/or large SVs. This is particularly useful when short-read sequencing indicates the presence of an SV and can be overlaid with long-read sequencing or OGM data to verify findings.
2. Realignment to the T2T-CHM13 reference genome and pangenome, taking advantage of reduced false-positive SV calling and resolved structural errors.^52131^,^133^
3. Leveraging joint-calling of SVs in large cohorts when applying minor allele frequency and case–control analysis approaches. Where possible, the trio and mode of inheritance filtering should be integrated into the analysis.
4. Careful selection of the most appropriate SV caller(s), and the use of multiple ‘ensemble’ SV callers for the most relevant and reliable results.^140^
5. Standardisation and reproducibility of SV calling pipelines to ensure high recall of SVs between batches and cohorts.

## Conclusion

IPN genetics has advanced remarkably over the past 10 years. MPS technologies have significantly impacted the provision of answers for patients with IPN, however, those families that remain genetically unsolved are a key research focus as we push the frontiers of human genomics. This review has highlighted strategies likely to help the IPN research community identify remaining disease-causing variants in novel genes, re-classify VUSs and overcome the rate-limiting steps of genomic data interpretation and functional validation. More extensive open data sharing and collaborative efforts will be crucial for sustained advances in IPN disease gene discovery and diagnostics. Genetic diagnoses are crucial for understanding disease pathobiology and guiding treatment development. The new tools for sequencing, mapping and analysis will achieve diagnoses for many more patients with IPN in the next 10 years.

## supplementary material

10.1136/jnnp-2024-333436 online supplemental file 1
