# Supplementary material for: Genetics of inherited peripheral neuropathies and the next frontier: looking backwards to progress forwards
Source: J Neurol Neurosurg Psychiatry. 2024 May 14;95(11):e333436. doi: 10.1136/jnnp-2024-333436 (PMC11503175; doi:10.1136/jnnp-2024-333436)
Supplement: online supplemental file 1 [file jnnp-95-11-s001.docx]

**The Genetics of Inherited Peripheral Neuropathies and the Next Frontier: Looking Backwards to Progress Forwards.**

Jevin M Parmar, Nigel G Laing, Marina L Kennerson, Gina Ravenscroft

## ***Supplemental Material***

## **Supplementary Table 1. List of 73 genes and two genetic loci associated with IPN that have been published since 2012.** Genes and loci were included in the table if they (1) had not previously been associated with any disease, (2) were previously associated with another disease, or (3) were previously reported for IPN, but, since 2012, have been newly associated with a different form of IPN. Genes and loci we consider to be associated with ultra-rare IPN are shaded in light-blue. Adapted from the 2023 gene table of neuromuscular disorders, which includes a full list of IPN-associated genes.(9)

| **DISEASE NAME** | **MOI** | **DISEASE SYMBOL (OMIM)** | **CHROMOSOME** | **GENE SYMBOL (OMIM)** | **PROTEIN** | **VARIANT TYPE** |
| --- | --- | --- | --- | --- | --- | --- |
| **Autosomal dominant CMT1 (AD-CMT1)** | | | | | | |
| Charcot-Marie Tooth disease, demyelinating type 1G | AD | CMT1G  (618279) | 8q21.13 | ***PMP2***  (170715) | Peripheral myelin protein 2 | SNV |
| Charcot-Marie-Tooth neuropathy | AD/  de novo | CMT1I  (619742) | 12q23.3 | ***POLR3B***  (614366) | Polymerase III, RNA, Subunit B | SNV |
| Charcot-Marie-Tooth neuropathy | AD |  | 6q21.31 | ***ITPR3***  (147267) | Inositol 1,4,5triphosphate receptor, type 3 | SNV |
| **Autosomal recessive CMT1 (AR-CMT1 or CMT4)** | | | | | | |
| Charcot-Marie Tooth disease, type 4B3 | AR | CMT4B3  (615284) | 22q13.33 | ***SBF1***  (603560) | SET-binding factor 1 | SNV, indel |
| Charcot-Marie Tooth disease, type 4K | AR | CMT4K  (616684) | 9q34.2 | ***SURF1***  (185620) | Surfeit 1 | SNV, indel |
| Charcot-Marie-Tooth disease | AR |  | 14q32 | ***AHNAK2***  (608570) | Ahnak  nucleoprotein 2 | SNV |
| Charcot-Marie-Tooth disease related to  CNTNAP1 | AR |  | 17q21.2 | ***CNTNAP1***  (602346) | Contactin-associated protein 1 | SNV |
| **Intermediate CMT (CMTi)** | | | | | | |
| Charcot-Marie-Tooth disease, dominant intermediate A | AD | CMT2GG  (606483) | 10q24.1-q25.1 | ***GBF1***  (603698) | Golgi-specific brefeldin-A resistance factor 1 | SNV |
| Charcot-Marie-Tooth disease, dominant intermediate F | AD | CMTDIF  (615185) | 3q28.33 | ***GNB4***  (610863) | Guanine nucleotide-binding protein, beta-4 | SNV, indel |
| Charcot-Marie-Tooth disease, dominant intermediate G | AD | CMTDIG  (617882) | 8p21.2 | ***NEFL***  (162280) | Neurofilament, light polypeptide 68kDa | SNV |
| Charcot-Marie-Tooth disease, intermediate | AD |  | 1p21.2-p13.3 | ***CFAP276* (*C1orf194*)**  (618682) | Chromosome 1 open reading frame 194 | SNV |
| Charcot-Marie-Tooth disease, intermediate | AD |  | 1p13.3 | ***SARS1***  (607529) | Seryl-tRNA synthetase 1 | SNV |
| **Autosomal dominant CMT2** | | | | | | |
| Charcot-Marie-Tooth disease, type 2Q | AD | CMT2Q  (615025) | 10p14 | ***DHTKD1***  (614984) | Dehydrogenase E1 and transketolase domain-containing 1 | SNV |
| Charcot-Marie-Tooth disease, type 2U | AD | CMT2U  (616280) | 12q13.3 | ***MARS1***  (156560) | Methionyl-tRNA synthetase 1 | SNV |
| Charcot-Marie-Tooth disease, type 2V | AD | CMT2V  (616491) | 17q21.2 | ***NAGLU***  (609701) | N-acetyl-alpha-glucosaminidase | SNV |
| Charcot-Marie-Tooth disease, type 2W | AD | CMT2W  (616625) | 5q31.3 | ***HARS1***  (142810) | Histidyl-tRNA synthetase 1 | SNV |
| Charcot-Marie-Tooth disease, type 2Y | AD | CMT2Y  (616687) | 9p13.3 | ***VCP***  (601023) | Valosin-containing protein | SNV, splice site |
| Charcot-Marie-Tooth disease, type 2Z | AD | CMT2Z  (616688) | 22q12.2 | ***MORC2***  (616661) | MORC family CW-type zinc finger 2 | SNV |
| Charcot-Marie-Tooth disease, axonal, type  2CC | AD | CMT2CC  (616924) | 22q12.2 | ***NEFH***  (162230) | Neurofilament Protein, Heavy Polypeptide | SNV, indel |
| Charcot-Marie-Tooth disease, axonal, type  2DD | AD | CMT2DD  (618036) | 1p13.1 | ***ATP1A1***  (182310) | ATPase, Na^+^/K^+^ transporting, alpha-1 polypeptide | SNV, indel |
| Charcot-Marie-Tooth disease, axonal, type 2FF | AD | CMT2FF  (619519) | 1q23.2 | ***CADM3***  (609743) | Cell adhesion molecule 3 | SNV |
| CMT2 related to *KIF5A* | AD |  | 12q13.3 | ***KIF5A***  (602821) | Kinesin family member 5A | SNV, indel, SV |
| Early onset axonal neuropathy with sensory ataxia | AD | CMT2 | 1q13.5 | ***DGAT2***  (606983) | Diacylglycerol O-acyltransferase 2 | SNV |
| Charcot-Marie-Tooth disease, axonal, related to *BAG3* | AD |  | 10q26.11 | ***BAG3***  (603883) | BCL2-associated athanogene 3 | SNV |
| Charcot-Marie-Tooth disease, axonal, related to *JAG1* | AD | CMT2 | 20p12.2 | ***JAG1***  (601920) | Jagged 1 | SNV |
| Charcot-Marie-Tooth disease, axonal, related to *NOTCH2NLC* | AD | CMT2 | 1q21.2 | ***NOTCH2NLC*** (618025) | Notch2 N-terminal-like protein | STR expansion |
| **Autosomal recessive CMT2** | | | | | | |
| Charcot-Marie-Tooth disease, axonal, type 2B2 | AR | CMT2B2  (605589) | 19q13.33 | ***PNKP***  (605610) | Polynucleotide kinase 3-prime phosphatase | SNV, indel |
| Charcot-Marie-Tooth disease, axonal, type  2EE | AR | CMT2EE  (618400) | 2p23.3 | ***MPV17***  (137960) | MPV17, mouse, homolog of | SNV, splice region |
| Charcot-Marie-Tooth disease, axonal, related to *DNAJB2* | AR |  | 2q35 | ***DNAJB2***  (604139) | DnaJ/Hsp40 homolog, subfamily B, member 2 | SNV, indel, splice site, CNV |
| Neuromyotonia and axonal neuropathy, autosomal recessive | AR | NMAN | 5q23.3 | ***HINT1***  (601314) | histidine triad nucleotide binding protein 1 | SNV |
| Charcot-Marie-Tooth disease, recessive intermediate, C | AR | CMTRIC  (615376) | 1p36.31 | ***PLEKHG5***  (611101) | Pleckstrin homology domain- and RhoGEF domain containing, family G5 | SNV, indel |
| Charcot-Marie-Tooth disease, recessive intermediate, D | AR | CMTRID  (616039) | 12q24.31 | ***COX6A1***  (602072) | Cytochrome c oxidase subunit 6a1 | Indel, splice region |
| Charcot-Marie-Tooth disease, type 2R | AR | CMT2R (615490) | 4q31.3 | ***TRIM2***  (614141) | Tripartite motif-containing protein 2 | SNV, indel |
| Charcot-Marie-Tooth disease, type 2S | AR | CMT2S (616155) | 11q13.3 | ***IGHMBP2*** (600502) | Immunoglobulin mu-binding protein 2 | SNV, indel, splice site |
| Charcot-Marie-Tooth disease, type 2T | AR, AD | CMT2T (617017) | 3q25.2 | ***MME***  (120520) | Membrane metalloendopeptidase | SNV, indel, splice region |
| Charcot-Marie-Tooth disease, type 2X | AR | CMT2X (616668) | 15q21.1 | ***SPG11***  (610844) | SPG11 vesicle trafficking associated Spatacsin | SNV, indel |
| Early-onset axonal Charcot-Marie-Tooth with ataxia | AR | AOA4 (616267) | 19q13.33 | ***PNKP***  (605610) | Polynucleotide kinase 3’-phosphatase | SNV, indel |
| Charcot-Marie-Tooth disease, axonal | AR |  | 10q22.1 | ***SGPL1***  (603729) | Sphingosine-1 phosphate lyase 1 | SNV |
| Charcot-Marie-Tooth disease, axonal; related to SCO2 | AR |  | 22q13.33 | ***SCO2***  (604272) | Cytochrome c oxidase assembly protein 2 | SNV |
| Charcot-Marie-Tooth disease, axonal; related to SACS | AR |  | 13q12.12 | ***SACS***  (604490) | Sacsin | SNV, indel, SV |
| Sorbitol dehydrogenase deficiency with peripheral neuropathy (CMT2 AR) | AR | SORDD (619912) | 15q21.1 | ***SORD***  (182500) | Sorbitol dehydrogenase | SNV, indel |
| Charcot-Marie-Tooth disease, axonal | AR |  | 21q22.3 | ***MCM3AP*** (603294) | Minichromosome maintenance 3-associated protein | SNV, indel |
| Charcot-Marie-Tooth disease, axonal | AR |  | 12q13.3 | ***B4GALNT1*** (601823) | Beta-1,4-N-Acetyl-galactosaminyl transferase 1 | SNV, indel |
| Charcot-Marie-Tooth disease, axonal | AR |  | 19p13.11 | ***MYO9B***  (602129) | Myosin IXB | SNV, indel |
| Charcot-Marie-Tooth disease, axonal | AD/  de novo |  | 1q25.3 | ***DHX9***  (603115) | DExH-Box helicase 9 | SNV, indel |
| **X-linked CMT (CMTX)** | | | | | | |
| Charcot-Marie-Tooth neuropathy, X-linked recessive, 3 | XR | CMTX3  (302802) | Xq27.1 | 78kb insertion of  Chr8 8q24.3 | 78kb inter-  chromosomal  insertion | SV |
| Charcot-Marie-Tooth neuropathy, X-linked 4 (Cowchock syndrome) | XR | COWCK  (310490) | Xq26.1 | ***AIFM1***  (300169) | Apoptosis-inducing factor, mitochondria-associated, 1 | SNV |
| Charcot-Marie-Tooth disease, X-linked dominant, 6 | XD | CMTX6 (300905) | Xp22.11 | ***PDK3***  (300906) | Pyruvate dehydrogenase kinase, isoenzyme 3 | SNV |
| **Distal hereditary motor neuropathy (dHMN)** | | | | | | |
| Distal hereditary motor neuronopathy | AR |  | 5q23.3 | ***HINT1***  (601314) | Histidine triad nucleotide-binding protein | SNV |
| Neuronopathy, distal  hereditary motor, type IID | AD | HMN2D (615575) | 7p14.3 | ***FBXO38*** (608533) | F-box only protein 38 | SNV |
| Distal spinal muscular  atrophy, type VB | AD,  AR | HMNS5B (614751) | 2p11.2 | ***REEP1***  (609139) | Receptor expression-enhancing protein 1 | SNV, splice site |
| Dominant distal hereditary motor neuropathy | AD | dHMN | 16q22.1 | ***AARS1***  (601065) | Alanyl-tRNA sythetase 1 | SNV |
| Neuronopathy, distal hereditary motor, type IX | AD | HMN9 (61772) | 14q32.2 | ***WARS1***  (191050) | Tryptophanyl-tRNA synthetase 1 | SNV |
| Spinal motor neuropathy |  |  | 11q23.2 | ***RBM7***  (612413) | RNA-binding motif protein 7 | SNV |
| Distal motor neuropathy related to *SYT2* | AD |  | 1q32.1 | ***SYT2***  (600104) | Synaptotagmin 2 | SNV |
| Distal hereditary motor neuropathy |  | DHMN1 | 7q34–q36.2 | 1.35 Mb insertion of  Chr7 7q36.3 | 1.35 Mb intra-  chromosomal  insertion (inverted) | SV |
| Distal motor neuropathy | AD |  | 9q34.11 | ***SPTAN1***  (182810) | Spectrin, alpha, nonerythrocytic 1 | SNV |
| Distal hereditary motor neuropathies | AD |  | 10q24.32 | ***GBF1***  (603698) | Golgi-specific brefeldin-A resistance factor 1 | SNV |
| Peripheral neuropathy, autosomal recessive, with or without impaired intellectual development | AR | PNRIID (618124) | 21q22.3 | ***MCM3AP***  (603294) | Minichromosome maintenance 3-associated protein | SNV, indel |
| Neuropathy, hereditary motor, with myopathic features | AR | HMNMYO (619216) | 1p36.33 | ***VWA1***  (611901) | Von Willebrand factor A domain-containing protein 1 | SNV, indel, STR expansion |
| Progressive neuropathy  (with ichthyosis, contractures) | AR |  | 9q21.2 | ***PSAT1***  (610936) | Phosphoserine Aminotransferase 1 | SNV |
| Neuronopathy, distal hereditary motor, autosomal recessive 2 | AR | HMNR2  (605726) | 9p13.3 | ***SIGMAR1***  (601978) | Sigma non-opioid intracellular receptor 1 | SNV, splice site |
| Neuronopathy, distal hereditary motor, autosomal recessive 9 | AR | HMNR9  (620402) | 16p12.3 | ***COQ7***  **(601683)** | Coenzyme Q7 hydroxylase | SNV, splice site |
| **Hereditary sensory and autonomic neuropathy (HSAN)** | | | | | | |
| Hereditary sensory and autonomic neuropathy, type IID | AR | CIP (24300) | 2q24.3 | ***SCN9A***  (603415) | Sodium channel, voltage-gated alpha subunit | SNV, indel |
| Hereditary sensory and autonomic neuropathy type VI | AR | HSAN6 (614653) | 6p12.1 | ***DST***  (113810) | Dystonin | SNV, indel |
| Neuropathy, hereditary sensory and autonomic, type VII | AD | HSAN7 (615548) | 3p22.2 | ***SCN11A***  (604385) | Sodium channel, voltage-gated alpha subunit | SNV, splice site |
| Neuropathy, hereditary sensory and autonomic, type VIII | AR | HSAN8 (616488) | 9q34.12 | ***PRDM12*** (616458) | PR Domain-containing protein 12 | SNV, indel, splice site, STR expansion |
| Neuropathy, hereditary sensory, type IF | AD | HSN1F (615632) | 11q13.1 | ***ATL3***  (609369) | Atlastin GTPase 3 | SNV |
| Absence of pain, Congenital | AR |  | 22q11.21 | ***CLTCL1***  (601273) | Clathrin, heavy polypeptide-like 1 | SNV |
| Marsili syndrome (insensitivity to pain, congenital, AD) | AD | MARSIS (147430) | 14q11.2 | ***ZFHX2***  (617828) | Zinc finger homeobox 2 | SNV |
| Episodic pain syndrome, familial 3 | AD | FEPS3 (615552) | 3p22.2 | ***SCN11A***  (604385) | Sodium channel, voltage-gated alpha subunit | SNV |
| Polyneuropathy with erythromelalgia | AR |  | 1q25.3 | ***NMNAT2*** (608701) | Nicotinamide nucleoside adenyltransferase 2 | SNV |
| Sensory neuronopathy | AR |  | 1q44 | ***COX20***  (614698) | Cytochrome c Oxidase Assembly Factor COX20 | SNV, splice site |
| Cerebellar ataxia, neuropathy, and vestibular areflexia syndrome | AR | CANVAS (614575) | 4p14 | ***RFC1***  (102579) | Replication factor C Subunit 1 | SNV, STR expansion |
| **Other complex neuropathy syndromes** | | | | | | |
| Giant axonal neuropathy-2 | AD | GAN2 (610100) | 1q23.2 | ***DCAF8***  (615820) | DDB1- and CUL4-associated factor 8 | SNV |
| Complex motor and sensory axonal neuropathy plus microcephaly and cerebral dysgenesis | AR |  | 14q32.2 | ***VRK1***  (602168) | Vaccinia related kinase 1 | SNV |
| Neuronal intranuclear inclusion diseases | AD | 603472 | 1q21.2 | ***NOTCH2NLC*** (618025) | Notch2 N-terminal-like protein | STR expansion |
| Hereditary peripheral neuropathy (CMT?) | AD |  | 17q25.1 | ***NHERF1***  ***(SLC9A3R1)***  (604990) | NHERF family PDZ scaffold protein 1 | SNV |
| Neurodevelopmental disorder with microcephaly, impaired language and gait abnormalities | AR, AD | NEDMILG (619091) | 18q21.31 | ***NARS1***  (108410) | Asparaginyl-tRNA Synthetase 1 | SNV, indel |
| Axonal neuropathy and motor dysfunction, with speech delay and intellectual disability | AR,  de novo |  | 14q21.3 | ***NEMF***  (608378) | Nuclear export mediator factor | SNV, indel, slice site |
| Neurodegeneration, childhood-onset, stress-induced, with variable ataxia and seizures | AR | CONDSIAS  (618170) | 1p34.3 | ***ADPRS***  ***(ADPRHL2)***  (610624) | ADP-riboslyserine hydrolase | SNV, indel, splice site |

AD, autosomal dominant; AR, autosomal recessive; CMT1, demyelinating Charcot-Marie-Tooth neuropathy; CMT2, axonal Charcot-Marie-Tooth neuropathy; indel, insertion/deletion; MOI, mode of inheritance; SNV, single nucleotide variants; STR, short tandem repeat; SV, structural variant; XR, X-linked recessive
